# α_1_-Adrenergic Receptors in Neurotransmission, Synaptic Plasticity, and Cognition

**DOI:** 10.3389/fphar.2020.581098

**Published:** 2020-09-29

**Authors:** Dianne M. Perez

**Affiliations:** The Lerner Research Institute, The Cleveland Clinic Foundation, Cleveland, OH, United States

**Keywords:** adrenergic receptor, G-protein coupled receptor, cognition, neurotransmission, synaptic plasticity

## Abstract

α_1_-adrenergic receptors are G-Protein Coupled Receptors that are involved in neurotransmission and regulate the sympathetic nervous system through binding and activating the neurotransmitter, norepinephrine, and the neurohormone, epinephrine. There are three α_1_-adrenergic receptor subtypes (α_1A_, α_1B_, α_1D_) that are known to play various roles in neurotransmission and cognition. They are related to two other adrenergic receptor families that also bind norepinephrine and epinephrine, the β- and α_2_-, each with three subtypes (β_1_, β_2_, β_3_, α_2A_, α_2B_, α_2C_). Previous studies assessing the roles of α_1_-adrenergic receptors in neurotransmission and cognition have been inconsistent. This was due to the use of poorly-selective ligands and many of these studies were published before the characterization of the cloned receptor subtypes and the subsequent development of animal models. With the availability of more-selective ligands and the development of animal models, a clearer picture of their role in cognition and neurotransmission can be assessed. In this review, we highlight the significant role that the α_1_-adrenergic receptor plays in regulating synaptic efficacy, both short and long-term synaptic plasticity, and its regulation of different types of memory. We will also present evidence that the α_1_-adrenergic receptors, and particularly the α_1A_-adrenergic receptor subtype, are a potentially good target to treat a wide variety of neurological conditions with diminished cognition.

## Introduction

**Figure 1:**
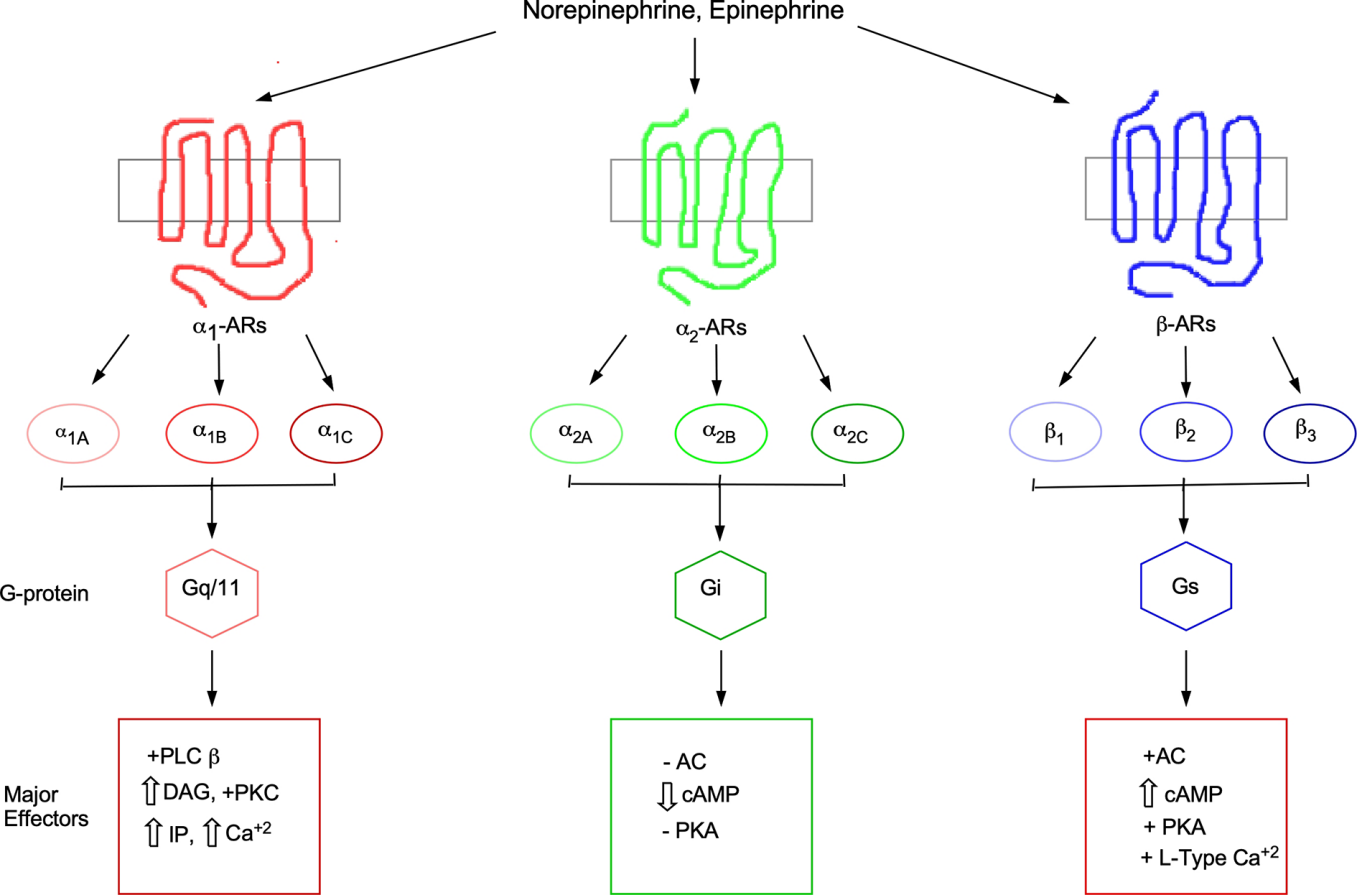
Adrenergic receptor family, subtypes, and signaling cascades. AC, adenylate cyclase; AR, adrenergic receptor; Ca, calcium; cAMP, cyclic adenosine-3',5'-monophosphate; DAG, diacylgycerol; IP, inositol phosphates; L-type Ca, L-type calcium channel; PLC, phospholipase C; PKA, protein kinase A; PKC, protein kinase C.

Raymond Ahlquist in 1948 (Ahlquist, 1948) first introduced the concept of different types of receptors called adrenergic receptors (ARs) which are activated by the same catecholamines, epinephrine (Epi) and norepinephrine (NE), but displayed opposite phenotypes in the body. He assigned them the subtypes of α and β. After the initial classification of α and β subtypes, decades of subsequent characterization in tissues during the 1980s further subdivided the α_1_-ARs into the α_1A_- and α_1B_-AR subtypes based upon pharmacological data. Using a series of several ligands in competition binding assays, the α_1A_-AR subtype was shown to display a 10–100-fold higher binding affinity compared to the α_1B_-AR subtype for a distinct series of ligands (Morrow and Creese, 1986). Subsequent cloning of the receptors confirmed this pharmacological distinction (Cotecchia et al., 1988; Laz et al., 1994; Perez et al., 1994). A few years later, another receptor was cloned that displayed novel pharmacology from the previous two subtypes and was named the α_1D_-AR (Perez et al., 1991). This classification of α_1_-ARs subtypes was approved by the IUPHAR Adrenergic Receptor Subcommittee in 1995 (Hieble et al., 1995). There exists a total of three AR families and nine subtypes (α_1A_, α_1B_, α_1D_, α_2A_, α_2B_, α_2C_, β_1_, β_2_, and β_3_) that display similar binding affinities but evoke different physiological effects for the same endogenous catecholamines. Signaling selectivity is achieved through the coupling to different G-proteins and effector systems in both temporal and spatial settings (**Figure 1**). There exists a modest number of selective agonists and antagonists to the different AR subtypes. The commercially available ligands that display at least a 10-fold in selectivity between the most related α_1_- versus α_2_-AR subtypes are shown in **Table 1**. Various ligands have often been employed as tools in research studies to dissect physiologies and signaling between the ARs but has often led to conflicting results, particularly the use of phentolamine which is not discriminating between the α_1_- versus α_2_-AR subtypes. Selectivity of at least 100-fold is required to significantly discriminate between the subtypes which none are commercially available except for the β_2_-AR agonists, salmeterol and formoterol. These two β_2_-AR agonists display 1,000-fold selectivity over the β_1_-AR by virtue of their ability to bind to the lipophilic transmembrane domains and increasing duration of action and are used clinically as bronchodilators to treat asthma (Lindén et al., 1996; Baker et al., 2015).

**Table 1:** Selective Agonists and Antagonists of the Alpha-Adrenergic Receptor Subtypes.

AR Subtype	Selective Agonists(> 10-fold)	Selective Antagonists(> 10-fold)
α_1_ > α_2_, β α_1_, α_2_ > β β > α_1_, α_2_ α_2_ > α_1_, β	Phenylephrine Isoproterenol Clonidine	Prazosin, terazosin Phentolamine Propranolol Rauwolscine
α_1A_-AR	Cirazoline A61603	+Niguldipine 5-Methylurapidil WB4101
α_1B_-AR	None	None
α_1D_-AR	None	BMY7378
α_2A_-AR	Oxymetazoline	BRL 44408
α_2B_-AR	None	Imiloxan
α_2C_-AR	None	JP1302

## α_1_-AR Localization in the Brain

All nine AR receptors are expressed in the brain (Hertz et al., 2010). Localization of the specific α_1_-AR subtypes throughout the brain has been difficult to ascertain because of the lack of tools for their assessment. Commercially available antibodies to many G-protein coupled receptors (GPCRs), and particularly the α_1_-ARs, have too low of avidity for localization and *in vivo* studies (Jensen et al., 2009). This is because of the sparseness of selective epitopes on the extracellular surface of the receptors and their endogenous expression levels which are typically in the low femtomolar range, rendering only the most avid antibodies the ability to distinguishing their true signal from background (Webb et al., 2013).

Due to the lack of avid antibodies, initial localization studies used labeled oligonucleotides that showed distinct distribution of the α_1B_-and α_1D_-AR subtypes in the rat brain (McCune et al., 1993; Pieribone et al., 1994). However, these short DNA sequences are likely not specific enough and could not achieve a high degree of specific activity, resulting in low sensitivity and specificity. Early autoradiography studies used non-selective ligands that could not distinguish the α_1_-ARs subtypes but did indicate that α_1_-ARs are abundant throughout the rat brain (Unnerstall et al., 1985). In later studies, ^3^H-prazosin was used to first label all α_1_-AR subtypes, then competed off with the α_1A_-AR selective antagonist, WB4101 and then label compared with ^3^H-WB4101 alone (Blendy et al., 1990). Here, the α_1B_-AR was prominent in the thalamus, lateral amygdaloid nuclei, and cortical laminar areas. The α_1A_-AR was prominent in the entorhinal cortex, amygdala but with more widespread distribution than the α_1B_-AR. The α_1A_-AR was also present in the cortex but in a homogenous and not laminar distribution that the α_1B_-AR indicated. However, this study was limited in scope because only a select coronal section of the brain was analyzed and sensitivity was low due to the use of tritium (Blendy et al., 1990). Much later, *in situ* hybridization studies using the full-length cDNA sequence confirmed that the α_1_-AR subtypes are indeed abundant and differentially expressed in the rat brain (Domyancic and Morilak, 1997). However, while hybridization studies are sensitive, they are not quantitative because they do not detect the protein, only nucleic acids. Radioligand binding of dissected tissue also offers high sensitivity and these studies indicated discreet brain domains in humans with high α_1_-AR content in the hippocampus and prefrontal cortex with the lowest expression in the caudate and putamen (Shimohama et al., 1986).

Eventually the development of transgenic and knock-out (KO) mouse models of the α_1_-AR subtypes were made and, in addition, mouse models with the α_1_-ARs tagged with either an enhanced green fluorescent protein (EGFP), endogenous promoter-driven expression of EGFP, or placement of β-galactosidase gene to KO the receptor. Using this transgenic-tagged approach, the α_1A_- and α_1B_-ARs was shown to exhibit similar expression patterns in the CNS, though the relative abundance was different (Papay et al., 2004; Papay et al., 2006). Both α_1_-AR subtypes are expressed in the amygdala, hippocampus, cerebellum, cortex, hypothalamus, midbrain, and spinal areas. Both α_1_-AR subtypes were also found in the same cell types such as neurons, interneurons, progenitors, and stem cells (Papay et al., 2004; Papay et al., 2006; Gupta et al., 2009). Using the α_1D_-AR KO, ^3^H-prazosin, and comparing radioligand receptor binding to normal wild-type mice, there was only modest loss of radioligand binding in the cortex, olfactory bulb, and CA1/CA3 hippocampus indicating low amounts of the α_1D_-AR subtype in the brain (Sadalge et al., 2003). Using transgenic KO mice for the individual α_1_-AR subtypes in conjunction with ligand binding studies (i.e. based upon the loss of binding thereof), the relative distribution of α1-ARs in the brain has been estimated to be composed of ~55% α1A (Rokosh and Simpson, 2002), 35% α1B (Cavalli et al., 1997), and 10% α1D (Tanoue et al., 2002).

Interestingly, the α_1A_- or α_1B_-ARs were not found in brain vascular cells or adult astrocytes using these mouse models, despite previous studies using primary and cell culture lines indicating expression of α_1_-ARs. α_1_-ARs are highly abundant in vascular smooth muscle in the periphery. This disparity can be explained in two ways. First, while a large fragment of the endogenous promoter (~4kb) was used to drive systemic expression of the α_1_-ARs in the transgenic mouse models used for localization studies, a limiting factor is that there may have been missing distant promoter regions that are responsible for expression of the subtypes in astrocytes and the brain vasculature. There are also studies which indicate that the α_1_-AR abundance and receptor subtype switches when tissue is processed into cells in culture or when the cells are immortalized (Schwarz et al., 1985; Ishac et al., 1992; Kajiyama and Ui, 1994). In studies using *in vivo* electrophysiology, α_1_-AR assignment to astrocytic function was based upon blockage using 200 μM prazosin, a dose 200-fold over its specificity for α_1_-ARs and could be blocking the α_2_-AR, which has a lower affinity for prazosin (Ding et al., 2013).

The binding and affinity of ligands for the α_1_-AR subtypes is similar between humans, mice and rats, showing no species differences in their pharmacological behavior. However, their localization in the brain may be different (Palacios et al., 1987; Zilles et al., 1991; Szot et al., 2005). While there are similar expression of α_1_-ARs in the thalamus and cerebral cortex between rodents, pigs, and humans, the expression of α_1_-ARs may be higher in the human hippocampus with α_1A_-AR subtype mRNA expressed in dentate gyrus and α_1D_-AR subtype mRNA expressed in the CA1-3 regions (Palacios et al., 1987; Szot et al., 2005). However, these studies used non-selective ligands and could not discriminate the subtypes or used mRNA localization which does not detect protein levels. Using the single cell polymerase chain reaction technique, the α_1A_-AR subtype alone was localized in the rat CA1 interneurons (Hillman et al., 2005). In additional studies using phenylephrine and the α_1A_-AR selective antagonist WB4101, α_1A_-ARs also functionally regulated the interneuron by depolarizing and subsequently releasing GABA and somatostatin (Hillman et al., 2005; Hillman et al., 2007). Using the α_1_-AR transgenic mouse models tagged with the EGFP, the α_1A_-AR expressed its highest densities in the CA1, CA3, and dentate gyrus of the mouse hippocampus and the hypothalamus (Papay et al., 2006) while the α_1B_-AR appeared most highly expressed in the cerebral cortex (Papay et al., 2004). Using both the EGFP transgenic and KO mouse models of the α_1A_- and α_1B_-AR subtypes, the α_1A_-AR subtype alone was also found to regulate the CA1 hippocampal interneurons (Jurgens et al., 2009), suggesting that the hippocampus has high α_1A_-AR content.

Radioligand binding studies of discreet brain domains in humans also indicate high α_1_-AR content in the hippocampus and prefrontal cortex cognitive domains (Shimohama et al., 1986) which were confirmed in later studies (Palacios et al., 1987; Szot et al., 2005), and in agreement with the transgenic mouse localization studies. The high, presumably α_1A_-AR content in human hippocampus suggests that therapeutics designed to enhance cognition through stimulation of the α_1A_-AR subtype may be translatable to human disease.

The α_1A_-AR subtype alone also regulates neurogenesis in the mouse hippocampus. The α_1A_- but not α_1B_-AR transgenic mice that systemically overexpress constitutively active receptors which are continually signaling, increased BrdU incorporation into the subgranular and subventricular zones (Gupta et al., 2009; Jurgens et al., 2009; Collette et al., 2010). Wild-type normal mice also increased BrdU incorporation when given the α_1A_-AR selective agonist, cirazoline, and maintained elevated levels for at least 14 days of chase, indicating that BrdU was incorporated into stem cells which divide very slowly (Gupta et al., 2009). α_1_-AR stimulation of adult neurospheres or co-localization studies using transgenic mice with EGFP-tagged α_1A_-ARs indicated that α_1B_- and α_1A_-ARs are expressed on neural and glial progenitors (Gupta et al., 2009). The EGFP-tagged α_1B_ -ARs in the transgenic mouse model was not expressed on stem cells but was expressed on progenitors in the rostral migratory pathway. However, the transgenic α_1B_-AR mice did not display evidence of increasing neurogenesis (Gupta et al., 2009) as assessed by BrdU incorporation. The regulation of neurogenesis by the α_1A_-AR may play a role in its synaptic plasticity, regulation of cognition, and therapeutics designed to activate this receptor subtype may provide some repair to the neurodegeneration that occurs in Alzheimer’s Disease.

## Neurophysiology of Norepinephrine

The NE system originates primarily in the locus coeruleus in the mammalian central nervous system (CNS) where NE is synthesized and released. From this area, different and diffuse projections of NE-releasing neurons disperse throughout the CNS to innervate the hippocampus, spinal cord, prefrontal cortex, cerebellum, thalamus, cortex, and amygdala (Iversen et al., 2009). The distribution of afferents to many key structures suggests a critical regulatory role of NE in the CNS. The NE system can modulate a number of functions such as learning and memory (Nguyen and Connor, 2019), but also depression and anxiety, sleep and arousal, brain development, motor activity, sensory information processing such as pain or touch, and to increase neurogenesis (Ordway et al., 2007a; Gupta et al., 2009). NE can cause repetitive firing and spike adaptation in the cerebral cortex, an essential function in controlling the excitability of neurons in the CNS.

Disruption in the NE system is also involved in a number of neurological diseases including Alzheimer’s disease (AD), epilepsy, attention-deficit disorder, Parkinson’s disease, depression, schizophrenia, and posttraumatic stress disorder (Ordway et al., 2007b). α1-ARs play a key role in several neurological systems and several neurological diseases that are associated with the NE system. Specificity in the NE signaling system is achieved from the nine AR subtypes even though they bind NE with very similar affinities. This is because AR subtypes couple to different G-proteins and effector proteins (**Figure 1**) that are temporally and spatially expressed in varying tissues throughout the brain and periphery that result in discrete expression patterns, signal transduction pathways, and physiological regulations.

## α_1_-AR Regulation of Synaptic Efficacy

The assignment of general neurological functions mediated by specific α_1_-AR subtypes is limited due to the lack of commercially available subtype-selective ligands with at least 100-fold selectivity between the subtypes (**Table 1**). α_1_-ARs are expressed on motor (Shao and Sutin, 1992), pyramidal neurons (Lazzaro et al., 2010; Santana et al., 2013; Zhang et al., 2013), sensory (Xie et al., 2001; Nicholson et al., 2005), Purkinje (Crepel et al., 1987; Herold et al., 2005; Schambra et al., 2005), and multiple types of interneurons (Bergles et al., 1996). The α_1_-ARs invoke both excitatory and inhibitory functions through postsynaptic and presynaptic mechanisms usually involving phospholipase C, protein kinase C (PKC), and/or calcium (**Figure 1**). α_1_‐AR activation can increase the firing frequency of pyramidal and somatosensory neurons of the rat visual cortex through a PKC signaling pathway (Mouradian et al., 1991; Kobayashi et al., 2008) and at presynaptic terminals to increase the inhibition of rat Purkinje neurons through phospholipase C-mediated release of calcium (Herold et al., 2005). Presynaptic α_1_-ARs can enhance glutamate or acetylcholine release to increase their excitation *via* a PKC or calcium pathway in the prefrontal cortex (Mouradian et al., 1991; Marek and Aghajanian, 1996; Chen et al., 2006; Velásquez-Martinez et al., 2012; Luo et al., 2014) or to prime excitatory synapses (Gordon and Bains, 2003). PKC is known to be involved in the phosphorylation of synaptic proteins or enhancing calcium sensitivity involved in the process of exocytosis of the vesicles (Shimazaki et al., 1996; Stevens and Sullivan, 1998; Hilfiker and Augustine, 1999; Wu and Wu, 2001). There is abundance evidence that α_1_-AR activation can modulate GABA-mediated inhibition in various and diverse brain regions (Mouradian et al., 1991; Marek and Aghajanian, 1996; Alreja and Liu, 1996; Bergles et al., 1996; Marek and Aghajanian, 1996; Kawaguchi and Shindou, 1998; Croce et al., 2003; Braga et al., 2004; Dumont and Williams, 2004; Herold et al., 2005; Hirono and Obata, 2006; Lei et al., 2007; Hillman et al., 2009; Kobayashi et al., 2009; Yuan et al., 2009; Salgado et al., 2011). The mechanism of α_1_-ARs stimulation of GABA release has been ascribed to a decrease in cellular resting conductance (McCormick et al., 1991; Bergles et al., 1996), but may also involve an increase in calcium or PKC signaling.

α_1_-ARs may also affect non-neuronal functions and modulate synaptic transmission as they may be expressed in astrocytes (Shao and Sutin, 1992; Bekar et al., 2008) and Bergmann glial cells, a specialized astrocyte in the cerebellum (Kulik et al., 1999). a_1A_-AR mRNA was expressed in astrocytes and NG2^+^-oligodendrocyte progenitors, but the α_1B_-AR mRNA was not present in any freshly isolated glial cells from the mouse cerebral cortex (Hertz et al., 2010). Progenitors and differentiated rat oligodendrocytes in culture functionally expressed only the α_1A_-AR subtype as assessed by its ability to increase inositol phosphate generation which was blocked by α_1A_-AR selective antagonists, 5-methylurapidil and WB4101 (Khorchid et al., 2002). Using transgenic mice expressing EGFP-tagged α_1A_-ARs, α_1A_-AR expression was found in NG2^+^-oligodendrocyte progenitors but not in mature oligodendrocytes *in vivo* (Papay et al., 2006). As mentioned earlier, interpretation of α_1_-AR expression in tissue culture cells is limited because α_1_-AR protein abundance changes and receptor subtype switches when tissue is processed into cells for culture or when the cells are immortalized (Schwarz et al., 1985; Ishac et al., 1992; Kajiyama and Ui, 1994).

Astrocytes are involved in neuro-glial communication to regulate the homeostasis in the brain and synaptic efficacy by controlling ion concentrations and removing excess released glutamate *via* transporters to prevent toxicity (Verkhratsky et al., 2019). α_1_-ARs expressed in astrocytes and Bergmann glia invoke a calcium flux thought to be important for neurotransmitter release and synaptic plasticity (Gordon et al., 2009; Ben Achour et al., 2010; Ben Achour and Pascual, 2010). The LC releases NE throughout the cortex and cerebellum in a phasic manner when responding to novelty, startle, or sensory input (Aston-Jones et al., 1991) and caused widespread Ca^+2^ signaling in cortical astrocytes that appeared to be regulated solely by the α_1_-AR in awake and startled but not sedated mice (Ding et al., 2013). In a similar manner, α_1_-AR activation on astrocytes in the mouse ventral periaqueductal grey were sufficient to increase arousal by promoting glutamate transmission (Porter-Stransky et al., 2019). The Ca^+2^ transients in Bergmann glial cells in locomotion-induced mice were also blocked by an α_1_-AR but not a β-AR antagonist (Kulik et al., 1999; Paukert et al., 2014). Therefore, α_1_-AR mediated calcium release in astrocytes may be the regulator of the principle astrocytic function of neuro-glia communication and particularly during sensory stimuli.

## Short-Term Synaptic Plasticity

Synaptic plasticity is a change in the strength or efficacy of a synapse and is commonly believed to be part of the cognitive process. The changes that occur during short-term plasticity can last from milliseconds to seconds and are usually associated with short bursts or ripples in activity causing transient changes in calcium in the presynaptic cleft (Zucker and Regehr, 2002). Synaptic plasticity declines with age and is associated with neurodegenerative disorders, such as Alzheimer’s Disease (Bach et al., 1999). α_1_-AR activation can slow or stop the normal spontaneous discharges of the CA3 pyramidal neurons to the hippocampal CA1 subfield (Scanziani et al., 1993; Rutecki, 1995). Stimulation of α_1_-ARs can also abruptly suppress the generation of sharp wave-ripple complexes in hippocampal slices allowing for rapid interruption of activity, such as those needed when the synchronized hippocampal activity needs to be switched into attention-related activity for processing new information (Ul Haq et al., 2012). This correlated synchronized activity leads to synaptic modifications that increase synaptic strength or plasticity (Buzsaki, 1989).

Glutamatergic plasticity is increased through α_1_-AR stimulation in a cooperative mechanism with corticotropin-releasing factor by enhancing inositol tri-phosphate mediated calcium release (Tovar-Díaz et al., 2018). This form of NMDA-dependent synaptic plasticity was demonstrated on ventral tegmental area dopamine neurons and promoted α_1_-AR mediated drug-associated learning (Tovar-Díaz et al., 2018) and increased motor activity (Goertz et al., 2015). Transgenic mice overexpressing constitutive active α_1A_-ARs increased basal synaptic transmission and short-term plasticity as assessed by paired-pulse facilitation (PPF) at the mouse CA3 and CA1 synapse (Doze et al., 2011). PPF is a change in a paired stimulus of a synapse when observed under a short period of time (i.e milliseconds) and is a form of synaptic enhancement. The second evoked excitatory postsynaptic potential is enhanced during PPF when it is followed immediately after the first evoked excitatory postsynaptic potential (Foster and McNaughton, 1991) and is used as an indirect measure of the probability of neurotransmitter release. Changes in PPF that are associated with long-term potentiation (LTP) suggest a presynaptic mechanism (Schultz et al., 1994), as synapses that are facilitated or potentiated must increase neurotransmitter release. The above α_1A_-AR transgenic mouse model displayed both increased LTP and PPF, suggesting that the α_1A_-AR-mediated increased in short-term plasticity is mediated through a pre-synaptic mechanism (Doze et al., 2011), caused by an increase in calcium flux in the presynaptic zone which led to the release of neurotransmitter in the CA1 region. While there are many mechanisms to induce PPF, the primary mechanism appears to be through increased calcium flux in the synaptic cleft (Jackman and Regehr, 2017).

## Long-Term Synaptic Plasticity

NE through activation of ARs increases the strength of synaptic transmission at glutamatergic synapses and modifies the synapses *via* cAMP signals and protein synthesis to increase long-term plasticity occurring over minutes to hours in duration (Hopkins and Johnston, 1984; Dahl and Sarvey, 1989; Harley, 1991; Bröcher et al., 1992; Harley and Sara, 1992; Huang and Kandel, 1996; Bramham et al., 1997; Erickson et al., 1997; Katsuki et al., 1997; Thomas and Palmiter, 1997a; Thomas and Palmiter, 1997b; Thomas and Palmiter, 1997c; Izumi and Zorumski, 1999; Watabe et al., 2000; Walling and Harley, 2004; Maity et al., 2020). As the signals involves cAMP, most previous studies have concluded that the sole AR in mediating NE effects on long-term plasticity have been the β-ARs (Maity et al., 2015; Hansen and Manahan-Vaughan, 2015; Nguyen and Gelinas, 2018). However, α_1_-ARs have been shown to mediate increased cAMP generation particularly in brain tissue independent of β-AR effects (Huang and Daly, 1972; Schultz and Daly, 1973; Stone et al., 1986; Stone et al., 1987).

A type of long-lasting plasticity is long-term potentiation (LTP) and is considered a major mechanism of learning and memory and has been mostly studied in the hippocampus. α_1_-ARs may be an important receptor in inducing long-lasting synaptic plasticity in the hippocampus (Sirviö and MacDonald, 1999). α_1_-AR agonists promoted while α_1_-AR antagonists blocked LTP formation in the rat CA1 hippocampus (Izumi and Zorumski, 1999) and may be dependent upon synergistic interaction with the β-AR through cAMP formation (Pedarzani and Storm, 1996). The activation of α_1_- and β-ARs facilitated tetanus-induced LTP at the mossy-fibers of the hippocampus (Hopkins and Johnston, 1984; Huang et al., 1996). α_1_-AR stimulation that could be blocked with prazosin also increased LTP in the dentate gyrus when acquisitioned during the learning of an active-avoidance behavior (Lv et al., 2016). The α_1A_-AR transgenic mice that systemically overexpresses the constitutively active α_1A_-AR subtype significantly improved LTP at the mouse CA3-CA1 synapse (Doze et al., 2011). These α_1A_-AR mice also showed increased cognition using the Barnes maze and a multi-T dry maze, while the similar mouse model that systemically expressing constitutive active α_1B_-AR subtype did not increase cognition (Doze et al., 2011). These transgenic studies imply that the α_1A_-AR subtype may be responsible for LTP and the cognitive-enhancing effects of α_1_-ARs.

α_1_-ARs also induce LTP in the neocortex. Using neocortical slices, α_1_-ARs stimulated on astrocytes caused the exocytosis of ATP which initiated a subsequent burst of ATP-mediated synaptic currents from ATP receptors on the pyramidal neurons inducing LTP (Pankratov and Lalo, 2015). α_1_-AR involvement in increasing LTP in the neocortex through releasing gliotransmitters was confirmed using terazosin to block the response. This work does suggest that α_1_-ARs may be involved in glia-neuron regulation of synaptic activity and plasticity. Calcium is released in the astrocytes through α_1_-AR activation which then can cause vesicular exocytosis of ATP to bind and activate purinergic receptors located on the adjacent pyramidal neurons (Pankratov and Lalo, 2015). Calcium is reported to be a significant signaling pathway in its communication with neurons for synaptic plasticity and LTP (Pascual et al., 2005; Guerra-Gomes et al., 2018).

α_1_-ARs also induced LTP in the prefrontal cortex (PFC) and is associated with increased cognition. Impaired α_1_-AR function following lesion in the ventral hippocampus decreased glutamatergic synaptic plasticity within the PFC and resulted in PFC-related cognitive dysfunction (Bhardwaj et al., 2014). This mechanism occurred through PKC‐dependent pathways in rat medial PFC (Luo et al., 2014) and required interaction with both glutamate release and N‐type Ca^2+^ channels (Luo et al., 2015b). α_1_-ARs also induce LTP in the ventral tegmental area on dopamine neurons through NMDA-mediated glutamatergic transmission utilizing a cooperative mechanism with corticotropin releasing factor to co-stimulate inositol triphosphate mediated calcium signaling (Tovar-Díaz et al., 2018).

Long-term depression (LTD) is also a form of long-term plasticity. Decreases in synaptic strength contribute to learning and memory functions by increasing flexibility in the synapse to store information (Heynen et al., 1996). LTD has been implicated in forms of learning and memory other than spatial memory such as the facilitation by exposure to novel objects (Manahan-Vaughan and Braunewell, 1999). Moreover, novelty exposure reversed LTP in the hippocampus (Xu et al., 1998). These findings suggest a correlation between LTD and novelty detection during learning and indicate that both LTD and LTP may impart different forms of synaptic information during spatial learning (Kemp and Manahan-Vaughan, 2004).

α_1_-ARs induced LTD at excitatory CA3–CA1 synapses in the rat hippocampus even when β-ARs were inhibited and 85% of the NE innervation was depleted by degeneration with the neurotoxin DSP-4 (Dyer-Reaves et al., 2019). α_1_-AR-mediated LTD proceeded through the ERK signaling pathway in hippocampal pyramidal neurons (Vanhoose et al., 2002; Scheiderer et al., 2008) and possessed characteristics of a novel form of synaptic plasticity (Hebb, 1949). This Hebbian-dependent LTD required coincident presynaptic and postsynaptic NMDAR activity (Scheiderer et al., 2004) and is independent of the “classical” LTD, induced by repetitive low frequency (1 Hz) synaptic stimulation (Mulkey and Malenka, 1992). The mechanism also involves postsynaptic activation of the α_1_-AR as the PPF ratio did not change (Scheiderer et al., 2004). In addition, there are reports that LTD required both co-activation of α_1_- and β-ARs (Katsuki et al., 1997) in addition to NMDA (Scheiderer et al., 2004) and the M1 muscarinic receptor (Scheiderer et al., 2008).

Serotonin neurons in the dorsal raphe nucleus regulate arousal and the modulate the response to stress (Joëls and Baram, 2009). Postsynaptic α_1_-AR activation using phenylephrine induced an inward current and LTD of the glutamate synapses on these serotonin neurons which was blocked using prazosin (Haj-Dahmane and Shen, 2014). Stress due to chronic restraint inhibited postsynaptic α_1_-AR mediated LTD on presynaptic glutamate synapses by downregulating the expression of the presynaptic CB1 cannabinoid receptor but had no effect on the α_1_-AR -mediated inward current. The lack of effect of stress on the α_1_-AR induced inward current suggests that chronic stress did not downregulate or desensitize the α_1_-ARs on these neurons. Synaptic plasticity may be induced *via* α_1_-AR-mediated LTD by increasing the expression of synaptic proteins (Abumaria et al., 2006). Activation of α_1_-ARs is also associated with inducing LTD on glutamate synapses in the visual cortex by postsynaptic mechanisms which alter the function of the α-Amino-3-hydroxy-5-methyl-4-isoxazolepropionic acid (AMPA) receptor (Kirkwood et al., 1999), NMDA (Treviño et al., 2012), and phospholipase C activation of inositol triphosphate release (Choi et al., 2005). Other brain areas that show postsynaptic α_1_-AR activation of LTD include the bed nucleus of the stria terminus that relays processing of the reward pathways (McElligott and Winder, 2008; McElligott et al, 2010), and in the prefrontal cortex through ERK and NMDA pathways (Marzo et al., 2010; Bhardwaj et al., 2014),

## General Cognitive Functions

The α_1_-ARs have been long associated to play a role in cognition (Sirviö and McDonald, 1999); however, its extent was not well characterized because of the lack of subtype specific ligands and animal models. Some early studies suggested that α_1_-AR activation inhibit spatial memory and consolidation in primates but used very low numbers of animals or very high concentrations of ligands (Arnsten and Jentsch, 1997; Mao et al., 1999). There is another report that memory consolidation is inhibited by α_1_-AR activation in chicks (Gibbs and Summers, 2001) but this could be a species-related variable. NE is reported to have an inverted U-shaped dosage in the regulation of learning and memory (Baldi and Bucherelli, 2005) and is hypothesized to explain the mixed results of NE on memory. However, an alternate hypothesis is that high doses of NE cause desensitization and downregulation of the ARs, known to result in a negative feedback on its response and function (Rajagopal and Shenoy, 2018). As will be reviewed here, most other and later studies indicate that α_1_-AR activation facilitates memory, motor and motivational behavior, memory retention, and storage.

In more recent studies using the transgenic and KO mouse models, the α_1B_-AR KO (Cavalli et al., 1997) resulted in impaired spatial learning to novelty and exploration (Spreng et al., 2001). The α_1B_-AR KO mice also showed a decrease in memory consolidation and fear-motivated exploration (Knauber and Müller, 2000a). α_1D_-AR KO mice did not display changes in spatial and emotional learning as well as contextual fear conditioning (Sadalge et al., 2003). Transgenic mice harboring constitutively active α_1A_-ARs that are constitutively stimulated have enhanced learning and memory using several cognitive behavioral tests, while α_1A-_AR KO mice (Rokosh and Simpson, 2002) showed deficits compared to wild-type (WT) controls (Doze et al., 2011; Collette et al., 2014). In the same study, WT mice given a 2-month treatment of cirazoline in the drinking water, which is 50-fold selective agonist for the α_1A_-AR versus the α_1B_-AR subtype, also displayed increased learning and memory (Doze et al., 2011). In addition, the α_1A_-AR transgenic displayed increased cognition in a battery of electrophysiological tests, such as basal synaptic transmission, PPF, and LTP compared with WT mice, consistent with the increased cognition displayed through behavioral studies (Doze et al., 2011). Together, these studies suggest that both the α_1A_- and α_1B_-AR but not the α_1D_-AR are involved in learning and memory processes.

## Conditioned Fear Memory

Stressful events that result from fear or aversive experiences have been associated with NE activation. The BLA also regulates fear conditioning (LeDoux, 2000). There is high expression of the α_1A_-AR subtype in that region (Domyancic and Morilak, 1997; Papay et al., 2006). α_1A_-AR subtype activation stimulates GABA-mediated miniature inhibitory postsynaptic currents in the BLA (Braga et al., 2004), suggesting that α_1A_-ARs may regulate fear conditioning. Fear conditioning promoted the excitability of the BLA by decreasing GABAergic inhibition through α_1_-ARs (Skelly et al., 2017) suggesting that blockage of α_1_-ARs may promote fear conditioned memory in the BLA (Lazzaro et al., 2010; Bernardi and Lattal, 2012) and in the prefrontal cortex which impaired conditioned fear extinction after injection of the α_1_-AR antagonist prazosin (Do-Monte et al., 2010). A subsequent study indicated that there was no effect of prazosin on the accrual of fear memory or retrieval but fear that was already established during prazosin treatment was more readily extinguished due to effects on the initial learning phase of the trauma (Lucas et al., 2019). This suggests that prazosin treatment could be used as a prophylactic during newly acquired fear experiences to prevent extinction-resistance.

Prazosin has been used to treat conditions that involve the return of aversive memories, such as extinguishing trauma-induced nightmares and sleep problems commonly associated with post-traumatic stress disorder in veterans (Raskind et al., 2003; Boehnlein and Kinzie, 2007; Dierks et al., 2007; Raskind et al., 2007; Miller, 2008; Taylor et al., 2008) and appear at odds with the studies that indicate α_1_-AR blockage promotes fear conditioning. However, a randomized, double-blinded clinical trial indicated that prazosin had no effect on nightmares or sleep disorders in veterans (Petrakis et al., 2016), indicating that previous reports were anecdotal. There were, however, significant effects of prazosin on decreasing alcohol dependency (Petrakis et al., 2016), part of the reward memory system that α_1_-ARs activate (Wada et al., 2020) and this could account for some of the anecdotal occurrences. The study by Lucas et al. (2019) suggests that if prazosin was given before or during the trauma induced in the veterans, nightmares would be more readily extinguished, but not after the fear conditioning had already been established.

α_1_-AR blockage in enhancing fear conditioning memory is also in contrast to its general role of promoting memory enhancement. A recent study has shown differential effects of NE on fear conditioning depending if the release of NE is long lasting or transient and could explain some of the discrepancies in fear-induced memory versus memory storage or spatial memory. Transient or bursting NE activity from a simple startle response elevated calcium levels in cortical astrocytes through activation of α_1_-ARs but prolonged NE activity during a head-fixed conditioned fear response elevated cAMP which was driven through activation of β-ARs (Oe et al., 2020). This study would suggest that low vigilance or acute stress memories may be enhanced by α_1_-AR activation but high vigilance and chronic stress-induced memories such as fear conditioning is promoted mostly through β-AR activation. Indeed, during chronic stress situations, α_1_-AR mediated stimulation of GABAergic interneurons is inhibited (Braga et al., 2004).

## Spatial Memory

Spatial and associative learning is commonly used in rodent studies of long-term memory and have been hypothesized to be dependent upon the dorsal hippocampus (Mahmoodi et al., 2010). Active allothetic place avoidance (AAPA) is a type of spatial navigational learning. In hippocampus-dependent learning using the AAPA task, the combination of the α_1_-AR antagonist prazosin and the β-AR antagonist propranolol impaired spatial avoidance learning (Petrasek et al., 2010). Similar effects were observed when prazosin was combined with α_2_-AR antagonist, idazoxan (Stuchlik and Vales, 2008a) or the D_2_ antagonist, sulpiride (Stuchlik et al., 2008b). α_1_-AR stimulation in the CA1 region of dorsal hippocampus improved spatial memory (Puumala et al., 1998) and histamine-induced spatial learning in the Morris water maze test (Torkaman-Boutorabi et al., 2014) and using a touchscreen trial unique non-matching to location task (Hvoslef-Eide et al., 2015). The transgenic mice that overexpress a constitutively active α_1A_-AR improved spatial memory in the Barnes, Morris and multi-T type mazes (Doze et al., 2011). KO of the α_1A_-AR gene (Doze et al., 2011) or the α_1B_-AR (Spreng et al., 2001) showed spatial memory impairments in the Morris water maze (Doze et al., 2011), while α_1D_-AR KO mice did not show deficits in spatial learning (Sadalge et al., 2003), but they did show deficits in working memory and attention (Mishima et al., 2004).

Corticosteroids, including glucocorticoids, along with norepinephrine are the two systems in the body that mediate the stress response and cause the body to adapt (de Kloet et al., 2005). Corticosteroids can have effects on learning and memory *via* interacting directly with their own receptors to mediate transcriptional or non-transcriptional effects due to stress (Wang et al., 2014) or interact and crosstalk with norepinephrine and its receptors in the hippocampus, prefrontal cortex, and the basolateral nucleus of the amygdala (Krugers et al., 2012) to increase astrocytic calcium waves, gliotransmitters, and glutamate release (Simard et al., 1999; Parpura et al., 2011) to effect cognition (Hassanpoor et al., 2014; Pearson-Leary et al., 2016; LaLumiere et al., 2017). Corticosteroid receptors also co-localize with α_1_-ARs and may even directly regulate each other (Williams et al., 1997). Glucocorticoid release during stress can cause spatial memory deficits in males and do so by increasing the frequency and amplitude of IPSCs. α_1_-AR induced increase in IPSCs are even further stimulated when co-stimulated with a stress-released glucocorticoid (Hartner and Schrader, 2018). This effect required the pretreatment with the synthetic glucocorticoid, dexamethasone, to prime the cells to respond to α_1_-AR activation, suggesting that corticosteroids modulate the signaling pathways of α_1_-ARs to mediate effects on memory (Hartner and Schrader, 2018). These results suggest a potential mechanism for spatial memory deficits caused by NE during increased stress which may be mediated through the α_1_-AR and its interactions with corticosteroid hormone receptors.

## Spatial Working Memory

The PFC can regulate goal-directed or motivational-related behavior planning and attention processes (Robbins and Arnsten, 2009). Spatial working memory relies on the function of the PFC and is targeted by various therapeutics to treat cognitive dysfunction. The PFC is required for temporary information storage during the execution of complex tasks. NE innervates the PFC (Lewis and Morrison, 1989) from afferents from the locus coeruleus.

All three families of ARs (α_1_, α_2_, β) are expressed throughout the PFC (Nicholas et al., 1993; Pieribone et al., 1994). The α_2A_-AR subtype may play a role in PFC-dependent cognition (Wang et al., 2007) and along with β-ARs, α_1_-ARs are also required for spatial memory, as α_1_-AR agonists increase and antagonists inhibit the formation of working memory (Puumala et al., 1998) and promote both focused and flexible attention (Berridge and Spencer, 2016). The radial arm maze is a type of spatial working memory test which retention is improved upon α_1_-AR activation (Pussinen et al., 1997). Attentional set shifting is also a test of spatial working memory that is also dependent upon the PFC. When there are elevated levels of NE release presynaptic vesicles, rats improved performance in working memory. Under these conditions of elevated NE release, α_1_-AR but not β-AR blockers blocked the improvement in working memory using the attentional set shifting test (Lapiz and Morilak, 2006). The effects of PFC-infused with the α_1_-AR agonist phenylephrine improved spatial working memory in a location task in rats (Hvoslef-Eide et al., 2015).

Psychostimulants such as methylphenidate improve sustained memory through α_1_-ARs (Berridge et al., 2012). There is also an improvement in working memory with the cognitive-enhancing drug modafinil that is hypothesized to be mediated by α_1_-ARs since effects are blocked by prazosin (Duteil et al., 1990; Stone et al., 2002; Winder-Rhodes et al., 2010). Modafinil is a wake-promoting non-amphetamine neurochemical with complex properties that can directly stimulate cortical catecholamine levels and indirectly stimulate serotonin along with other neurotransmitters (Minzenberg and Carter, 2008; Chen et al., 2013; Scoriels et al., 2013). Together, these results suggest that α_1_-AR activation could be used to target enhancement of spatial working memory.

The mechanism of α_1_-AR regulation of PFC-mediated spatial working memory is likely due to increasing the release of glutamate from glutamatergic terminals within the PFC and promoting a persistent excitatory effect of pyramidal neurons (Marek and Aghajanian, 1999; Zhang et al., 2013; Bhardwaj et al., 2014). It is enhanced when the presynaptic α_1_-ARs are facilitated by postsynaptic α_2_-ARs inhibition of hyperpolarization-activated cyclic nucleotide-gated cation channels (Zhang et al., 2013) or post-synaptic *via* PKC-mediated enhancement of AMPA and NMDA excitatory currents (Luo et al., 2014).

In another study, phenylephrine also increased GABAergic transmission onto the pyramidal neurons in the medial PFC through inhibiting the interneuron inwardly rectifying potassium channels (Kirs), which caused the depolarization of the interneuron leading to an increased calcium influx through calcium channels (Luo et al., 2015a). The disruption of GABAergic transmission in the PFC can also produce impairments in working memory (Enomoto et al., 2011; Bañuelos et al., 2014). Therefore, α_1_-ARs may work to improve spatial working memory through both glutamatergic and GABAergic mechanisms.

## Reward Memory

The medial PFC is also involved in reward-related memories by receiving major dopamine (DA) neurotransmission from the ventral tegmental area (VTA), which then projects back onto the VTA and the nucleus accumbens in the forebrain. These networks play a prominent role in the reward circuitry (Tzschentke, 2000; Schultz, 2015).

The VTA receives major inhibitory GABAergic innervation from the nucleus accumbens as well as other areas in the brain (Jhou et al., 2009a; Jhou et al., 2009b) that controls their patterns in firing (Paladini and Tepper, 1999; Lobb et al., 2010) and contributes to burst activation and prolonged activity of DA neurons in the nucleus accumbens and prefrontal cortex (Paladini et al., 2003; Jhou et al., 2009a; Lobb et al., 2010; Morikawa and Paladini, 2011; Lohani et al., 2018). Since burst-firing can result in increased efficacy and enhanced neurotransmitter release in the presynaptic terminal (Fisher et al., 1997; Floresco et al., 2003), GABAergic regulation of DA bursting activity is one way to modulate reward-related memories. DA can cause the co-activation of α_1_-ARs (Leedham and Pennefather, 1986; Rey et al., 2001; Cornil et al., 2002; Zhang et al., 2004; Lazou et al., 2006; Lin et al., 2008) and α_1_-ARs are expressed on presynaptic terminals in the nucleus accumbens to regulate DA neurotransmission (Paladini et al., 2001; Cui et al., 2004; Mitrano et al., 2012).

DA neurons increased short-term burst firing in reaction to rewards while drugs that are addicting produce repetitive bursting (Covey et al., 2014; Keiflin and Janak, 2015). α_1_-ARs have also been shown to increase VTA-DA neurotransmission and induce burst firing (Grenhoff et al., 1993; Grenhoff and Svensson, 1993; Grenhoff et al., 1995; Paladini and Williams, 2004). Presynaptic α_1_-AR also facilitated glutamatergic inputs that affect VTA-DA neurotransmission (Velásquez-Martinez et al., 2012; Bocklisch et al., 2013) and participates in addiction-related effects (Cecchi et al., 2002; Jimenez-Rivera et al., 2006; Greenwell et al., 2009). α_1_-AR stimulation revealed a cooperative mechanism with corticotropin-releasing factor (CRF) on VTA neurons that increased NMDA receptor-mediated glutamatergic plasticity to induce the learning of cocaine-associated behavior (Tovar-Díaz et al., 2018). The CRF co-stimulated inositol triphosphate-mediated calcium release along with the α_1_-AR activation of these signals and blockage of the conditioning was suppressed by co-administration of both CRF and α_1_-AR blockers (Tovar-Díaz et al., 2018). α_1_-AR stimulation in the medial PFC increased cocaine craving, effects that were blocked by the α_1_-AR antagonist terazosin (Wada et al., 2020). α_1_-AR blockage by prazosin also decreased the motivational memory of nicotine (Forget et al., 2009). These results suggest that changes in the α_1_-AR signaling induced through drugs of abuse could be part of the neuromodulation occurring in the reward circuitry during the development of addicting behavior.

## Memory Consolidation, Storage, and Recall

The entorhinal cortex (EC) facilitates the neuronal connections and communication between the hippocampus and cortical areas that are required for consolidation and recall of memories (Haist et al., 2001; Squire et al., 2004; Dolcos et al., 2005; Steffenach et al., 2005). Alteration in communication between the principal cells and the interneurons in the EC is a mechanism for the ability to process spatial information which is disrupted when there are spatial memory deficits (Couey et al., 2013; Pastoll et al., 2013).

The EC expresses α_1_-ARs (Stanton et al., 1987) and is prominent in α_1A_-AR density (Papay et al., 2006). α_1_-ARs increased spontaneous inhibitory postsynaptic currents (IPSCs) in both frequency and amplitude when recorded from the principal neurons in the EC (Lei et al., 2007). DA facilitated the α_1_-ARs-mediated GABA release in the EC by inhibiting Kirs, a potassium channel which further depolarizes interneurons resulting in Ca^+^ influx *via* T-type Ca^+^ channels (Cilz et al., 2014). Inhibitory inputs are important for the activity in the EC. Changes in the signal-to-noise ratio of the inhibitory signals could alter the theta-nested gamma oscillations that are needed for spatial memory processing (Chrobak and Buzsaki, 1998; Quilichini et al., 2010; Colgin, 2015; Colgin, 2016).

α_1_-AR activation can enhance memory recall and consolidation. The α_1_-AR antagonist, prazosin, blocked the NE-facilitated effects of reconsolidation during fear conditioning (Gazarini et al., 2013). Using a discriminative avoidance task, α_1_-ARs were necessary for the consolidation both short-term and intermediate-term memory in the chick (Gibbs and Bowser, 2010). This mechanism was suggested to be mediated through an increased calcium release though astrocytic α_1_-ARs as effects were blocked with metabolic inhibitors for astrocytes (Gibbs and Bowser, 2010). Astrocytes, unlike neurons, mediate learning and memory upon glycogenolysis needed for the synthesis of glutamate (Gibbs et al., 2008; Newman et al., 2011).

NE regulation of the basolateral nucleus of the amygdala (BLA) is involved in the consolidation and storage of memory (Ferry and McGaugh, 2000). While the β-ARs are usually considered the main mechanism of NE’s effects on memory consolidation through the cAMP pathway (Ikegaya et al., 1997; Ferry and McGaugh, 1999), β-ARs and α_1_-AR may be needed together to enhance memory storage in the BLA. α_1_-AR blockage in the BLA decreased the stimulation of cAMP through a β-AR agonist or the increased effect of a synthetic cAMP analog on memory storage (Ferry et al., 1999a; Ferry et al., 1999b), while activation of α_1_-ARs can potentiate β-AR-mediated increases in cAMP formation in the BLA to enhance memory storage (Ferry et al., 1999a; Ferry et al., 1999b). Phenylephrine alone impaired memory retention in the BLA but when infused with the α_2_-AR antagonist yohimbine increased memory retention (Ferry and Quirarte, 2012) suggesting that the cross talk of activating presynaptic α_2_-ARs led to the memory impairing effects. KO mice that has the gene deletion for the α_1B_-AR indicated a decrease in latency in the passive avoidance test suggesting deficits in memory consolidation (Knauber and Müller, 2000b).

In the rat dorsal hippocampal CA1 regions, α_1_-AR activation reversed cannabinoid-induced amnesia (Moshfegh et al., 2011). Pre-test dorsal hippocampal intra-CA1 administration of the α_1_-AR agonist phenylephrine reversed the loss of memory during retrieval that was induced with the synthetic cannabinoid agonist, WIN55,212-2. Pre-test use of an α_1_-AR antagonist prazosin inhibited the WIN55,212-2 response (Moshfegh et al., 2011). There was a similar effect of α_1_-AR activation on reversing scopolamine-induced amnesia (Azami et al., 2010) and when agonists were administered before electroconvulsive shocks that decreased the induced amnesia and enhanced recall using a passive avoidance test (Anand et al., 2001).

## Olfactory Memories

The mammalian main olfactory bulb (MOB) receives major modulatory input from the locus coeruleus (McLean et al., 1989). MOB exhibits one of the highest densities of α_1_-ARs using autoradiography of the non-selective radiolabeled antagonist I^125^-HEAT (Jones et al., 1985) and specifically for the α_1A_-AR subtype (Domyancic and Morilak, 1997; Papay et al., 2016). Olfactory stimuli evokes NE release in the MOB and NE is an important signal in specific olfactory learning, memory, reward-motivated discrimination, and pheromonal regulation of reproductive/maternal behaviors through the excitation of mitral cells (Pissonnier et al., 1985; Sullivan et al., 1989; Kendrick et al., 1991; Sullivan et al., 1992; Rangel and Leon, 1995; Jiang et al., 1996; Brennan et al., 1998; Guérin et al., 2008; Mandairon et al., 2008; Eckmeier and Shea, 2014; Harvey and Heinbockel, 2018).

Both NE and the α_1_-AR agonist phenylephrine increased evoked activation of mitral cells that were also inhibited by the α1A-AR selective antagonist WB-4101 (Ciombor et al., 1999). The inward current caused by α_1_-ARs are mediated by decreased K^+^ conductance (Hayar et al., 2001). α_1_-AR are known to be a major effector of the NE released in the MOB and the resulting GABAergic inhibition on the mitral cells, increasing their excitation (Perez et al., 1987; Mouly et al., 1995; Ciombor et al., 1999; Nai et al., 2010) and particularly though the α_1A_-AR subtype (Ciombor et al., 1999; Zimnik et al., 2013). Transgenic mice that systemically overexpress constitutively active α_1A_-ARs or in normal mice given the α_1A_-AR selective agonist, cirazoline, increased adult neurogenesis as assessed though increased BrdU incorporation in the subventricular and subgranular zones and the number of neuronal progenitors migrating to the MOB (Gupta et al., 2009). The MOB receives and continually integrates newly generated neurons through neurogenesis all throughout adult life (Ming and Song, 2005). These neurons develop and integrate as GABAergic interneurons (Panzanelli et al., 2009; Valley et al., 2013). Sensory synaptic plasticity commonly occurs during dynamic increases in inhibition (Carcea and Froemke, 2013). Together, these results suggest that the α_1A_-AR subtype could be responsible for the various forms of learning and memory in the MOB through its ability to increase GABAergic interneuron inhibition of mitral cells.

These studies are opposite to what was shown in the accessory olfactory bulb located at the posterior region of the olfactory bulb. Presynaptic activation of α_1_-ARs increased GABA-induced miniature IPSCs frequency to increase the release of GABA from granule cells in the accessory olfactory bulb and decrease the excitability of mitral cells (Araneda and Firestein, 2006). α_2_-ARs are also present in the MOB but their activation suppressed IPSCs (Nai et al., 2009; Nai et al., 2010). NE effects on the MOB have been shown to be biphasic and can cause both excitation and inhibition of mitral cells (Okutani et al., 1998). Together, these results could suggest that way that α_1_-ARs regulate olfactory memories could be different in the accessory olfactory bulb versus the MOB.

## α_1_-ARs in Alzheimer’s Disease and Dementias

Pathology and degeneration of the neurons in the EC may also be contributors to AD progression (Hyman et al., 1984; Kotzbauer et al., 2001) and NE innervation in the medial EC is decreased in rodent models of AD (Chalermpalanupap et al., 2017; Rorabaugh et al., 2017). There is also sprouting of NE fibers in the hippocampus and PFC of subjects with AD, consistent with regeneration that is observed after neuronal loss (Szot et al., 2006). AD patients have elevated NE levels even during cell loss which is thought to be due to compensatory changes (Szot et al., 2006).

The LC is the provider of NE innervation (Compton et al., 1995; Aston-Jones, 2004), and modulates the synaptic efficiency needed for cognition (Harley, 1991; Kemp and Manahan-Vaughan, 2004; Scheiderer et al., 2004; Harley, 2007). Degeneration of the LC along with the first signs of tau pathology in AD has been well documented (Forno, 1966; Yamada and Mehraein, 1977; Zarow et al., 2003; Grudzien et al., 2007; Braak et al., 2011; Jucker and Walker, 2011; Arendt et al., 2015; Chalermpalanupap et al., 2017; Kelly et al., 2017; Theofilas et al., 2017). The hippocampus showed a decline in LTP with advancing age indicating an impairment in synaptic plasticity (Landfield and Lynch, 1977). Visual-induced memory loss in the perirhinal cortex during AD is also impaired through decreases in LTD in a mouse model of AD (Tamagnini et al., 2012). The study of Dyer-Reaves et al. (2019) indicated that α_1_-ARs induced LTD even when 85% of the NE innervation was lost through degeneration, suggesting that α_1_-AR agonists could be used as a treatment for the cognitive decline associated with AD that is due to neurodegeneration.

α_1_-ARs have been previously associated with AD, but there are no previous studies exploring the effect of AR agonists and antagonists in clinical studies, only assessed through changes in receptor density and mRNA. Changes in α_1_-AR function may contribute to aging process in the loss of memory function. There is a report that α_1_-AR density is upregulated in the aged mouse brain and improved passive avoidance learning, supporting a role for these receptors in age-related cognitive decline (Knauber and Müller, 2000b). Changes in PFC function can have effects on learning and memory (Brozoski et al., 1979) and is also an area of degeneration in AD (Poirel et al., 2018). The mRNA expression of the α_1A_-AR specifically was significantly decreased in the layers of the PFC in patients with AD with no changes in the α_2_-AR (Szot et al., 2007) and there is an α_1A_-AR polymorphism associated with AD (Hong et al., 2001). Overall α_1_-AR receptor density as assessed through radioligand binding in AD is also reduced by 25% (Shimohama et al., 1986). Age-related impairments in spatial memory using the Morris water maze in rats indicated that stimulation of the α_1_-AR improved cognition (Riekkinen et al., 1997). These results suggest that therapies to increase α_1_-AR signaling may be able to improve cognitive decline in AD.

After AD, vascular dementia is the second-most frequent form of dementia. Agonistic autoantibodies against the α_1_-ARs were found to be significantly present in 50% of people diagnosed with dementia, and in particular with those who also had heart disease (Karczewski et al., 2012a; Hempel et al., 2016; Thyrian et al., 2018). These autoantibodies bound to the first extracellular loop of the α_1A_-AR subtype, suggesting that this was the epitope used by the body to generate the autoantibodies (Karczewski et al., 2012a). The first extracellular loop of the α_1A_-AR would be able to confer specificity of the antibody to this subtype because the extracellular loops of the ARs are the least conserved between family members in their amino acid sequence. Cognitive function can be stabilized over a long period with the complete removal of the antibodies (Karczewski et al., 2018). Using animal models, it was demonstrated that α_1_-AR autoantibodies caused vascular impairment in the brain and induced a type of vascular dementia (Zhou et al., 2008; Karczewski et al., 2012a; Karczewski et al., 2012b; Pohlmann et al., 2014). While these results suggest that activation of α_1_-ARs may lead to disease progression, GPCR autoantibodies may be generated only after the disease is present, suggesting that the body is trying to compensate for the loss of the receptor function during the course of the disease. For example, agonistic autoantibodies against the angiotensin receptor induced vasoconstriction as seen for the agonist, angiotensin II, only in ischemic but not normal arteries (Lukitsch et al., 2012). Similar results were found when antagonistic autoantibodies of the β_2_-AR only inactivated the receptor during ischemic but not normal cell culture conditions in myocytes (Wallukat and Wollenberger, 1987). Therefore, agonistic autoantibodies against the α_1A_-AR may develop during AD to compensate for their loss in receptor density as documented by Szot et al. (2007) and Shimohama et al. (1986).

Interestingly, autoantibodies against the α_1_-AR were often found together with β_2_-AR autoantibodies in dementia patients (Karczewski et al., 2012a; Perez et al., 2019). The β_2_-AR autoantibodies were directed against the amino acid sequences in the second extracellular loop but could display both agonist and antagonistic effects (Mijares et al., 2000). It was postulated that receptor autoantibodies can act as an agonist when the α_1_ and β_2_-AR receptors dimerize and as an antagonist on the receptor monomer. While both α_1_- and β_2_-AR can form receptor homodimers, only the α_1B_-AR subtype was shown to not form heterodimers with the β_2_-AR (Stanasila et al., 2003). It could be speculated that the α_1A_-AR can form heterodimers with the β_2_-AR and autoantibodies against them can trans-inhibit their function, produce negative allosteric interactions, altered or new trafficking of signaling pathways, which are common effects with GPCR heterodimers (Haack and McCarty, 2011).

## Drug Development and Allosteric Modulators

A major function and limitation of the α_1_-AR subtypes are the ability to contract vascular smooth muscle which results in the regulation of blood pressure. α_1_-ARs efficiently couple to the G_q_ G-protein that activates the effector phospholipase C causing the release of inositol triphosphate (IP3) from membrane phospholipids. It is IP release that increases intracellular calcium that contracts smooth muscle causing increased blood pressure for agonists that activate this receptor (Heagerty et al., 1986; Huzoor-Akbar et al., 1989). This side effect of blood pressure regulation is a major reason why drug development essentially halted for this receptor. All of the commercially available ligands for the α_1_-AR have the potential to interact with the other subtypes and/or crossover to β-AR or α_2_-ARs. There are no existing ligands that demonstrate more than 100-fold selectivity between the α_1_-AR subtypes. Ligands with 50 to 100-fold selectivity are good research tools to dissect functions of the different subtypes, but not selective enough for therapeutics.

Allosteric modulators are currently being pursued in the pharmaceutical industry as the next wave of therapeutics to treat disease. There are several advantages to using allosteric modulators when compared to current orthosteric agonists (Christopoulos, 2002). The effects of allosteric ligands are saturating; once the allosteric sites are fully occupied by a drug, there is no further observed allosteric effect. In contrast, drugs that bind to the orthosteric site can have continuous effects and compete for occupancy determined by the relative concentrations of the two species (i.e. endogenous neurotransmitter vs. orthosteric drug). Therefore, there is a ceiling to the potential effects of an allosteric drug and can be given in high doses without the concern of causing additional side effects from overstimulating the system. Another advantage of allosteric drugs is their ability to selectively activate the receptor only in places in which the endogenous agonist is binding and signaling. This is achieved because allosteric modulators are often ligand-specific and signaling-biased in the conformational changes they induce (i.e. NE and cAMP specific). In addition, normal neurohumoral signaling involves the activation of nerves that release neurotransmitters in temporal and spatial settings. An allosteric modulator can cause its effects only when the endogenous agonist it specifically modulates is present. If neurotransmission is reduced, the allosteric drug would have little effect, even if the neurotransmitter is still present in the body. Furthermore, positive allosteric modulators may enhance signaling when receptors are degenerating since they potentiate or enhance existing signals. Allosteric drugs also could have greater receptor selectivity. The amino acids that contribute to allosteric binding sites are usually different from those that comprise the orthosteric binding site, which are usually the regions with highest conservation between receptor subtypes. For example, the amino acids that comprise the agonist binding pocket for ARs share a number of common residues. Therefore, while all the ARs may be stimulated by NE, only an α_1A_-AR positive allosteric modulator will enhance the α_1A_-AR NE-mediated effects, achieving subtype selective responses.

Conformational changes and signaling-bias, crucial benefits of allosteric modulators were recognized early for the α_1_-ARs. In the early 1980s it was recognized that the way phenethylamine agonists bound and signaled through the α_1_-AR was different from the way imidazoline agonists interacted (Ruffolo and Waddell, 1982). While most imidazolines have better selectivity for α_2_-ARs (Ruffolo and Waddell, 1983), key differences in the substitutions can render imidazolines α_1_-AR selective (Ruffolo et al, 1980; Hieble et al., 1986; Knepper et al., 1995). The α_1A_-ARs are preferentially activated by imidazolines (Minneman et al., 1994) and imidazolines have biased-signaling towards the cAMP response when compared to the primary pathway and blood pressure-inducing signaling of IP/Ca^+2^ even though they are not allosteric modulators (Evans et al., 2011; da Silva et al., 2017). α_1A_-AR imidazoline partial agonists have been shown to mediate functions that are uncoupled from blood pressure at low doses (Blue et al, 2004; Musselman et al., 2004). Based upon the hypothesis that α_1A_-AR stimulation would increase cognition, my laboratory developed a series of positive allosteric modulators that are not agonists and do not evoke an IP response on their own, but conformationally potentiate the cAMP response of NE (Perez et al., 2019). It is postulated that cAMP is the cognitive signal that induces memory formation through α-ARs (Ferry et al., 1999a; Ferry et al., 1999b) as well as for NE-mediated effects on memory (Liang et al., 1986; Liang et al., 1990; Introini-Collison et al., 1991; Katsuki et al., 1997; 82-84; 65). These α_1A_-AR positive allosteric modulators are currently in pre-clinical studies.

There are three published reports of allosteric modulation at α_1_-ARs, and all are described as negative allosteric modulators with no known clinically-useful application as of yet (Leppik et al., 2000; Sharpe et al., 2003; Campbell et al., 2017). Positive allosteric modulators have also been developed for the β_2_-AR (Ahn et al., 2018; Liu et al., 2019). While these modulators are selective for the β_2_-AR and display cooperative signals with agonists, they are not ligand-selective nor signaling-biased. However, all these studies give hope for the eventual development of allosteric modulators for the ARs that may be used to treat disease and specifically for AD.

## Concluding Remarks

There is substantial evidence that α_1_-ARs play an important role in synaptic efficacy and plasticity with effects on increasing learning and memory functions. There is no longer any controversy on their roles in cognition that was prevalent in the 1990s and early 2000s due to the use of nonselective ligands or using too high concentrations of ligands that would cross talk with other ARs. α_1_-ARs are expressed on a wide variety of neurons but also on glia and both contribute to its effects on cognition. α_1_-AR activation can improve conditioned fear, spatial, reward, olfactory, storage, recall and consolidation of memories. Consolidating and understanding the mechanisms involved in neurotransmission, learning, and memory may lead to new treatments in neurodegenerative diseases and age or disease-mediated cognitive decline. With the advent of allosteric drugs for GPCRs, α_1_-ARs that may become an effective treatment option in AD and other dementias without the unwanted side effects on blood pressure.

## Author Contributions

The author confirms being the sole contributor of this work and has approved it for publication.

## Funding

This work was supported by a grant from NIH RO1AG066627 and The Edward N. & Della L. Thome Memorial Foundation Awards Program in Alzheimer’s Disease Drug Discovery Research.

## Conflict of Interest

The author declares that the research was conducted in the absence of any commercial or financial relationships that could be construed as a potential conflict of interest.
